# Socioeconomic Status (SES) and Children's Intelligence (IQ): In a UK-Representative Sample SES Moderates the Environmental, Not Genetic, Effect on IQ

**DOI:** 10.1371/journal.pone.0030320

**Published:** 2012-02-01

**Authors:** Ken B. Hanscombe, Maciej Trzaskowski, Claire M. A. Haworth, Oliver S. P. Davis, Philip S. Dale, Robert Plomin

**Affiliations:** 1 Medical Research Council Social, Genetic and Developmental Psychiatry Centre, Institute of Psychiatry, King's College London, London, United Kingdom; 2 The Wellcome Trust Centre for Human Genetics, University of Oxford, Oxford, United Kingdom; 3 European Bioinformatics Institute, Wellcome Trust Genome Campus, Hinxton, Cambridge, United Kingdom; 4 Department of Speech and Hearing Sciences, University of New Mexico, Albuquerque, New Mexico, United States of America; The University of Queensland, Australia

## Abstract

**Background:**

The environment can moderate the effect of genes - a phenomenon called gene-environment (GxE) interaction. Several studies have found that socioeconomic status (SES) modifies the heritability of children's intelligence. Among low-SES families, genetic factors have been reported to explain less of the variance in intelligence; the reverse is found for high-SES families. The evidence however is inconsistent. Other studies have reported an effect in the opposite direction (higher heritability in lower SES), or no moderation of the genetic effect on intelligence.

**Methods:**

Using 8716 twin pairs from the Twins Early Development Study (TEDS), we attempted to replicate the reported moderating effect of SES on children's intelligence at ages 2, 3, 4, 7, 9, 10, 12 and 14: i.e., lower heritability in lower-SES families. We used a twin model that allowed for a main effect of SES on intelligence, as well as a moderating effect of SES on the genetic and environmental components of intelligence.

**Results:**

We found greater variance in intelligence in low-SES families, but minimal evidence of GxE interaction across the eight ages. A power calculation indicated that a sample size of about 5000 twin pairs is required to detect moderation of the genetic component of intelligence as small as 0.25, with about 80% power - a difference of 11% to 53% in heritability, in low- (−2 standard deviations, SD) and high-SES (+2 SD) families. With samples at each age of about this size, the present study found no moderation of the genetic effect on intelligence. However, we found the greater variance in low-SES families is due to moderation of the environmental effect – an *environment*-environment interaction.

**Conclusions:**

In a UK-representative sample, the genetic effect on intelligence is similar in low- and high-SES families. Children's shared experiences appear to explain the greater variation in intelligence in lower SES.

## Introduction

A key construct for understanding the interplay between nature and nurture is genotype-environment (GxE) interaction: Genes can have different effects on a phenotype depending on the environment, and environments can have different effects depending on genes [1], [2], [3], [4], [5], [6]. Twin and adoption studies divide the population variation in a trait, e.g. height, into fractions attributable to genetic and environmental factors. The net genetic contribution to population variation, i.e., what makes one person different from another, can be expressed as a *heritability* statistic (h^2^). However, if the effects of genes and environments do not simply “add up”, i.e., if there exists a GxE interaction, heritability will depend on the level of the moderating environment.

The education, occupation and income of parents – indices of the families' socioeconomic status (SES) – have been found to moderate the heritability of their children's intelligence [7], [8], [9], [10]. The most recent twin study in this area reported significant moderation of the genetic component of children's intelligence (IQ, or general cognitive ability, *g*) by their parents' SES [9]: a GxE interaction in which heritability of intelligence *increased* with SES. Focusing on early cognitive development, the study found an increasing heritability of the change in IQ between the ages of 10 months and 2 years as a function of SES. Although SES was measured as a continuous variable, the magnitude of genetic moderation found suggested an increase in the heritability of IQ from 5% in low-SES families (−2 standard deviations, SD), to 50% in high-SES families (+2 SD).

It is reasonable to consider the possibility that heritability of intelligence is higher in higher SES families because such families seem likely to provide more opportunities to realize differences in children's genetic potentials. Conversely, in lower SES families, genetic differences might be restrained by poverty. Two theories, the *bioecological model* [11] and the *environmental disadvantage hypothesis* [12], [13], predict this direction of GxE interaction effect – greater genetic contribution to IQ in high-SES families. It is important to note that these theories make predictions about how children will react to the environment they experience in the *real* world, but the interactions reported are statistical and model-dependent [4]. However appealing these reports may be, the moderating effect of SES is not consistently found. Several studies are either less conclusive [14], find no moderation of the heritability of IQ by level of SES [15], [16], or find a trend in the opposite direction – greater heritability of children's IQ in lower-SES families [17]. Table 1 summarises the previous studies.

**Table 1:** Gene-environment (GxE) Interaction Twin Studies of SES and Cognitive Measures.

Country	Number of pairs	Age	Analytical model	SES measure	Cognitive measure	GxE	Heritability
*[Study Ref.]*						*Higher h^2^ in higher SES*	*Low SES* – *High SES*
**UK** [17]	∼1000 MZ, ∼1000 DZ	4 years	Extended DF analysis	Parental education & occupation, and age of mother at birth of first child	Verbal factor	No	81%–49%^a^
					Non-verbal factor	No	21%–42%^a^
<$>\scale 80%\raster="rg1"<$> **Sweden** [7]	94 MZ, 229 DZ	12 years	Stratification and inspection of twin correlations	Parental education & occupation	Verbal test (opposites)	Yes	48%–76%
					Non-verbal test (logic)	Yes	21%–96%
**US** [15]	1774 MZ, 1429 DZ	16–30 years	**Continuous moderator**	Parental education	Armed Forces Qualification Test	No	56%–45%
**US** [8]	1909 (176 MZ, 347 DZ, 795 full-sib, 269 half-sib, 118 cousins, 204 unrelated)	16 years	Extended DF analysis	Parental education	Peabody Picture Vocabulary (verbal IQ)	Yes	26%–74%
<$>\scale 80%\raster="rg1"<$> **US** [14]	96 MZ, 69 DZ	10–15 years	Stratification and inspection of twin correlations	Parental education and occupation (census tract data)	Composite of 5 tests	No^e^	52%–50%
<$>\scale 80%\raster="rg1"<$> **US** [13]	503 Black pairs, 275 White pairs^d^	6–18 years	Stratification and inspection of twin correlations	Parental education and occupation(census tract data)	Composite of 5 tests	Yes	∼0%–27%
						Yes	∼0%–40%
**US** [9]	188 MZ, 562 DZ	10 months & 2 years	**Continuous moderator**	Parental education, occupation & income	Bayley Mental Development Index	Yes^c^	5%–50%
**US** [10]	114 MZ, 205 DZ	7 years	**Continuous moderator**	Parental education & occupation	WISC IQ	Yes	10%–72%
							(based on twin correlations from a median split - not the continuous moderator parameters)
**Netherlands** [16]	130 MZ, 144 DZ	26 & 49 years	**Continuous moderator**	Parental education	WAIS IQ	No	–b

<$>\vskip -3\scale 65%\raster="rg1"<$>indicates studies considered to have unreliable estimates based on small samples and/or non-standard zygosity assignment.

a 15% cut-offs for low and high SES (non-significant estimates for 25%, 33% and 50% cut-offs also reported in original paper).

b Not reported.

c GxE significant for change in mental ability from 1 to 2 years.

d No zygosity information; MZ and DZ twin correlations estimated from data from same-sex and opposite-sex twins.

e Results averaged over 5 tests and 2 ethnic groups.

**Figure 1:**
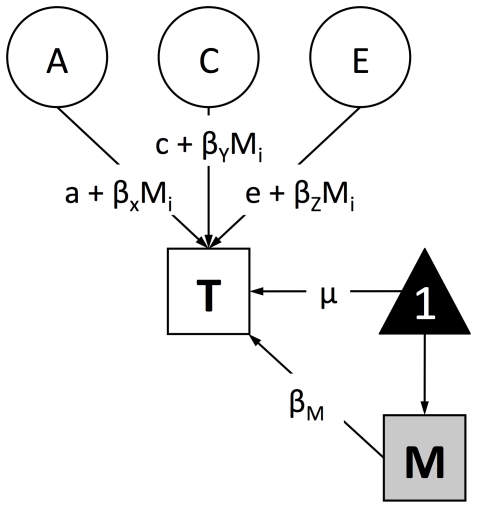
Continuous moderator model. The measured moderator (M) has a mediating or main effect (β_M_) on the trait (T), as well as a potential moderating effect on the variance components of the residual (after the main effect has been partialled out). A, C, E = additive genetic, shared environmental, and nonshared environmental variance components (of residual T); a, c, e = unmoderated elements of genetic, shared, and nonshared path coefficients; β_A_, β_C_, β_E_ = moderated elements of the genetic, shared, and nonshared path coefficients; M_i_ = measured moderator level for the ith twin pair (both twins in a pair have the same value for obligatorily-shared moderators like SES); μ = the mean of the trait (T); 1 = the constant by which μ is multiplied, values of the trait are given by *1μ+β_M_*.

At least three design differences could play a role in the inconsistent findings: first, statistical GxE interaction has been investigated with a variety of methods with different power to detect an interaction; second, the age range investigated has covered infancy (10 months, [9]) to adulthood (49 years, [16]) – age groups which may not be directly comparable; third, the samples have been drawn from different demographics (representing different points on the SES distribution), or different countries in which socioeconomic status may be more or less a factor for children's intelligence. Given the large range of ages studied and the variety of SES indices used, the present study set out to replicate the reported increasing heritability with increasing SES at each of eight ages from early childhood to adolescence in a large UK-representative sample by systematically applying the continuous moderator model [18]. The continuous moderator model can be used to measure potential SES moderation of the genetic and environmental influences typically found by the classic twin design (effects on the variance components of IQ), after accounting for main effects of the measured environment (effects on the mean level of IQ). The twin model typically divides the trait variance into additive genetic (A) and shared environmental (C) influences that explain twin similarity, and nonshared environmental (E) influences that explain twin differences. Figure 1 and the method section describe how the continuous moderator model incorporates moderation of each of these terms.

For several power-related reasons, the moderation of environmental factors (in particular experiences shared by children reared together - *shared environment*, C) may be particularly important in explaining the inconsistent reports of GxE interaction. The continuous moderator model, used by several of the studies investigating GxE interaction, has demonstrated low power to distinguish between moderation of the genetic (A) and shared environmental (C) variance components. Purcell [18] notes that specificity of the model is an issue – an observation made by the first study to report SES moderation of the heritability of IQ using this model ([10], p. 627): “Although the models indicate that the (*β_A_*, *β_C_*, and *β_E_*) interactions jointly contributed significant variance to differences in (IQ), the models were less able to distinguish which of the individual interactions with A, C, and E was most important.” (*β_A_*, *β_C_*, and *β_E_* represent SES moderation of the genetic, shared, and nonshared environmental influences on IQ.) Nonetheless, the full model, which simultaneously takes into account all influences on a trait (moderated and un-moderated, genetic and environmental), tends to recover the true parameter values in simulated data [18]. Regardless of which terms have been found to be significant and what decisions have been made about the presence or absence of particular moderating effects, because of the difficulty distinguishing between genetic and environmental moderation, estimates from the full model are preferable to those derived from a model in which individual terms have been fixed to zero.

A more general power consideration is that twin studies use the same information to estimate the genetic and shared environmental influence on a trait with the result that large samples are required to detect moderate shared environment [19]. Moreover, the relative contribution of the shared environment to population variation in a variety of traits including IQ has been shown to decrease with age [20], [21], [22].

Using a large population-based United Kingdom (UK) twin sample, with longitudinal data on IQ from infancy to adolescence, we aimed to address these age, population, and power concerns. We set out to replicate the finding that SES modifies the genetic effect on children's intelligence with three indices of SES: parental education and occupation measured when the twins were 18 months old; the same composite of education and occupation measured when the twins were 7 years old; and family income measured when the twins were 9 years old. The possibility that the environmental disadvantage hypothesis applies to academic achievement and reading measures has also been studied. However, because achievement and reading are quite different from IQ, and studies of them are no more conclusive about the presence or absence of GxE interaction, in the present study we choose to focus on IQ only. Given the inconsistency in the literature, we hypothesized that we would not find consistent GxE interaction from childhood to adolescence.

## Methods

### Participants

This study used as its sampling frame the ongoing Twins Early Development Study, TEDS [23], [24]. TEDS is a population-based longitudinal study of over 10,000 pairs of twins born in England and Wales in 1994, 1995, and 1996. Zygosity was assigned to the twins using a parent-rated instrument that yielded 95% accuracy when compared to zygosity established from DNA markers [25]; uncertainties were followed up with DNA marker testing. Comparison to census data from the Office of National Statistics indicates that the sample has remained reasonably representative of the United Kingdom population [26]. Ethical approval for the Twins Early Development Study has been provided by the King's College London ethics committee (reference: 05/Q0706/228). The parents of the twins provide informed written consent for each TEDS assessment.

The present study investigated the moderating role of parental SES on children's intelligence or IQ (measured as general cognitive ability, *g*) at ages 2, 3, 4, 7, 9, 10, 12, and 14. Analyses were performed on a subsample of 8716 twin pairs (2996 monozygotic (MZ); 5720 dizygotic (DZ)) for whom we had IQ data for at least one twin (at any one age), and with at least one index of SES. Subsets of these data were assessed at each age. In the analyses described below, we used all the available data with full-information maximum likelihood procedures.

### Measures

#### Socioeconomic status, SES

As indices of SES, we used parental education, occupation, and family income. We assessed parental education and occupation (mother's and father's highest educational qualification and job status) at first contact with the families, when the twins were 18 months old, and again when the twins were 7 years old; we assessed family income at age 9. To explore the possibility that the inconsistency in the literature is due to different measures of SES, we tested three indices: *SES index 1*, parental education and occupation acquired at contact (age 18 months); *SES index 2*, parental education and occupation acquired at age 7; and *SES index 3*, parental income assessed at age 9. All composites were created as a unit-weighted sum of the contributing scales, i.e., first mapped to a standard normal distribution with the rank-based van der Waerden transformation [27], then summed, and finally standardized again. The correlations between these three SES estimates are 0.77 for SES index 1 and 2, 0.55 for SES index 1 and 3, and 0.57 for SES index 2 and 3.

#### General cognitive ability, g

At all ages, a unit-weighted composite of verbal and nonverbal cognitive tests was used as an index of *g*. We mapped all verbal and nonverbal cognitive tests to a standard normal distribution [27], summed the contributing scales, and standardized the final *g* composite. This score was identical to a first principal component extracted from the balanced test battery.

#### Measures at Ages 2, 3, and 4

In early childhood, parent-administered tests and parent-reported observations were used to assess verbal and nonverbal cognitive abilities at each age. These measures have been validated against standard tests administered by a trained tester [28], [29].

Nonverbal performance: Nonverbal cognitive performance was assessed using age-appropriate versions of the Parent Report of Children's Abilities (PARCA; [28], [29]). The PARCA is an hour-long test comprising three types of parent-administered tasks: a “find the pair” task, a drawing task, and a matching task. Some items are novel; others are adapted from previously well-validated tests such as the McCarthy Scales of Children's Abilities [30] or the Bayley Scales of Infant Development (BSID-II; [31]). Together, the administered items are designed to assess number, shape, size, conceptual grouping and orientation skills. This parent-administered component is supplemented by a small number of parent report items anchored on concrete behaviors and requiring simple yes or no answers. Some of these items are novel; others are adapted from previously well-validated assessments such as the Minnesota Child Development Inventory (MCDI; [32]) and the Ages and Stages Questionnaires [33]. The complete PARCA, including novel and previously well-validated items, has been validated in an independent sample [29] and in the TEDS sample [28].

Verbal performance: The verbal component of the early childhood battery included vocabulary and grammar as assessed by parent reports for the CDI-III, an extension of the short form of the MacArthur Communicative Development Inventories: Words and Sentences [34]. The MCDI has been shown to have excellent internal consistency and test–retest reliability, as well as concurrent validity with tester-administered measures [34].

#### Measures at age 7

At age 7, verbal and nonverbal abilities were tested by telephone [35]. Prior to the telephone call, parents were sent a booklet of test items along with instructions indicating, for example, that the test booklet should not be opened prior to the telephone interview and that the twins should not be in the same room for the duration of the call. The booklet contained two tests of verbal cognitive abilities and two nonverbal tests. The verbal tests consisted of the Similarities subtest and the Vocabulary subtest from the Wechsler Intelligence Scale for Children (WISC-III-UK; [36]). The nonverbal tests were the Picture Completion subtest from the WISC-III-UK and Conceptual Grouping from the McCarthy Scales of Children's Abilities [30].

#### Measures at age 9

Nine-year-old participants received a test booklet containing two verbal and two nonverbal tests that, like the tests in early childhood, were administered under the supervision of the parent (guided by an instruction booklet rather than a telephone interview). The verbal tests comprised vocabulary and general knowledge tests adapted from the multiple-choice version of the WISC-III-UK [36]. The nonverbal tests included a Puzzle test adapted from the Figure Classification subtest of the Cognitive Abilities Test 3 (CAT3; [37]Smith, Fernandes, & Strand, 2001). The second nonverbal test was a Shapes test also adapted from the CAT3 Figure Analogies subtest that assesses inductive and deductive reasoning. Details are reported by Davis et al. [38].

#### Measures at age 10

Children at age 10 participated in web-based testing. Widespread access to inexpensive and fast internet connections in the UK has made online testing an attractive possibility for collecting data on the large samples necessary for genetic research. The advantages and potential pitfalls of data collection over the Internet have been reviewed [39]. For older children, most of whom are competent computer users, it is an interactive and enjoyable medium. Through adaptive branching, it allows the use of hundreds of items to test the full range of ability, while requiring individual children to complete only a relatively small number of items to ascertain their level of performance. In tests where it is appropriate, streaming voiceovers can minimize the necessary reading. In addition, the tests can be completed over a period of several weeks, allowing children to pace the activities themselves, although they are not allowed to return to items previously administered. Finally, it is possible to intersperse the activities with games. All of these factors help to maintain children's engagement with the tests. Participants at age 10 were tested on two verbal tests: WISC-III-PI Multiple Choice Information (General Knowledge) and WISC-III-PI Vocabulary Multiple Choice [36]. Two nonverbal reasoning tests were also administered: WISC-III-UK Picture Completion [36] and Raven's Standard Progressive Matrices [40]. Details are reported in Haworth et al. [41].

#### Measures at Age 12

At age 12 we again used Web-based assessment of general cognitive ability. The tests administered were updated versions of the same tests used at age 10, with the addition of more difficult age-appropriate items. We administered two verbal ability tests: WISC-III-PI Information Multiple Choice (General Knowledge) and WISC-III-PI Vocabulary Multiple Choice [42]. We also administered two nonverbal tests: Raven's Progressive Matrices [40] and WISC-III-UK Picture Completion [36].

#### Measures at Age 14

At age 14 we measured general cognitive ability with one verbal and one non-verbal Web-based test. The verbal test used was WISC-III-PI Vocabulary Multiple Choice [42]; the nonverbal test used was Raven's Progressive Matrices [40]. Both measures were the age-appropriate versions of those tests used at earlier ages.

The correlations between these eight IQ scores are shown in Table 2.

**Table 2:** Phenotypic correlations between IQ measures.

*Age*	*2*	*3*	*4*	*7*	*9*	*10*	*12*	*14*
***2***								
***3***	0.64							
	(0.62–0.66)							
***4***	0.54	0.69						
	(0.52–0.56)	(0.67–0.71)						
***7***	0.25	0.26	0.30					
	(0.22–0.28)	(0.23–0.29)	(0.27–0.33)					
***9***	0.19	0.23	0.26	0.43				
	(0.15–0.23)	(0.19–0.27)	(0.22–0.30)	(0.40–0.46)				
***10***	0.20	0.21	0.23	0.42	0.56			
	(0.16–0.24)	(0.17–0.25)	(0.19–0.27)	(0.38–0.45)	(0.53–0.59)			
***12***	0.16	0.20	0.24	0.45	0.52	0.59		
	(0.12–0.20)	(0.16–0.24)	(0.21–0.27)	(0.42–0.48)	(0.49–0.55)	(0.56–0.62)		
***14***	0.17	0.19	0.19	0.44	0.47	0.51	0.61	
	(0.12–0.22)	(0.14–0.24)	(0.15–0.23)	(0.40–0.48)	(0.43–0.51)	(0.47–0.55)	(0.58–0.64)	

Correlations are based on one randomly selected member of each twin pair.

## Statistical analysis

### Twin model fitting

The twin design compares the phenotypic resemblance of identical (monozygotic, MZ) twins to the phenotypic resemblance of non-identical (dizygotic, DZ) twins in order to partition the variance on a trait into sources of genetic and environmental variation. The coefficient of genetic relatedness is 1.0 between MZ twins, and on average 0.5 between dizygotic twins, who share 50% of their segregating alleles. The twin model attributes the similarity of reared-together twins to additive genetic (A) and shared environmental (C) factors, and the differences between them to nonshared environmental (E) factors [43]. By definition, co-twins in both MZ and DZ pairs are correlated 1.0 for C factors. The assumptions of the twin design and attempts to validate them are described in detail elsewhere [44].

Structural equation model fitting with full-information maximum-likelihood estimation provides a comprehensive way to estimate genetic and environmental sources of variation within traits and covariation between traits. We used the matrix optimization package OpenMx [45] in R (www.R-project.org; [46]) to fit structural equation models to the phenotypic covariance structure between twins. The fit of a particular model to the data is summarized by a fit statistic, negative two times the log likelihood (−2lnL); differences in −2lnL across different models distribute as chi-square (*χ* ^2^) which provides a goodness-of-fit test. The *χ* ^2^ can be converted to Akaike's information criterion (AIC; AIC = *χ* ^2^−2*df*; [47]), a measure of model fit relative to parsimony.

### Univariate GxE model

We used the basic continuous GxE model [18] to estimate the moderating effect of SES on IQ. This model allows the putative moderator to have a *main* effect on the trait, as well as a *moderating* effect on any or all of the residual A, C, and E components of the trait. Figure 1 summarizes the structural equation model for a single twin.

The mean of trait (T) is given by *μ + β_M_M*, where *β_M_* represents the phenotypic regression coefficient. The *main* effect of the measured environment (M) on the trait is assessed by estimating the value of *β_M_*. In the present study the trait is IQ and the moderator is SES. The residual variance in the trait is then partitioned into latent A, C and E components; the effect of each of these components on the trait is also expressed as a linear function of the moderator. For example, the additive genetic path coefficient is made up of both an unmoderated element (*a*) and a moderated element (*β_A_M_i_*), where *M*_i_ represents the family-wide moderator value for the *i*th twin pair. The significance of the *moderating* effect of SES is tested by asking whether *β_A_* is significantly different from zero. Likewise, the C and E path coefficients (*β_C_* and *β_E_* respectively) indicate the moderating effect of SES on the shared and nonshared environmental components of the residual variance in IQ and their significance is tested against zero.

One limitation of the basic GxE model is that it cannot detect potential moderation of any genetic variation in common between the measured environment and the trait, and SES is phenotypically correlated with IQ. It is well established that ‘environmental’ measures are to some extent heritable – a phenomenon known as genotype-environment correlation [2], [48], [49]. In the present study however, SES is the same for both members of a twin pair (they are children in the same household), so that the extent of genetic influence on SES cannot be assessed in our twin design. Nonetheless, any unmeasured genetic variation in SES that also explains variation in IQ is partialled out as part of the basic GxE model (and included in the *β_M_* term in the means model).

### Power estimation

We used exact data simulation with the continuous moderator model to estimate power to detect GxE moderation, and in particular, moderation of the latent genetic (A) component. For all power calculations we used the MASS [50] and OpenMx [45] packages, in the statistical computing environment R (www.R-project.org; [46]). For a range of sample sizes, effect sizes, a given genetic and environmental effect, a normally distributed moderator, and a specified moderation, we simulated data to which we fitted the basic continuous moderator model [18] and obtained a fit statistic, −2lnL. We then fitted a (constrained) model with the moderator term dropped, and calculated the difference in fit, Δ−2lnL which distributes as chi-square. We repeated this procedure 1000 times for each set of initial values, and plotted the distribution of chi-square statistics. Given that we simulated a significant non-zero moderation then dropped this term in the constrained model, the power to detect a particular effect size was the percentage of these replicates whose chi-square value was greater than 3.84 (the critical value for a 1df chi-square test, with significance value of p = 0.05).

In order to generate outcome data under continuous moderation, we first sampled *N* random values for MZ pairs and *N* for DZ pairs from a standard normal distribution. This was our obligatorily-shared moderator (SES in the present study). Then, for each level of the moderator we drew a single pair from a multivariate standard normal distribution. The variance-covariance matrix for each randomly sampled pair was specified by (the covariance structure of the basic continuous moderator model), MZ twin pairs
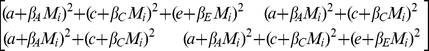
DZ twin pairs
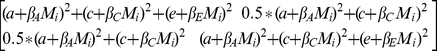
where *Mi* was the value of the moderator for the *i*th twin pair; a, c, and e are the unmoderated path coefficients; and *β_A_*, *β_C_*, *β_E_*, and are the moderated path coefficients. (R script available from authors upon request.)

## Results

The means, standard deviations, and analysis of variance by sex and zygosity for IQ at every age are presented in Table 3. There was no indication of any differences by zygosity or sex. In general, we find no significant effect of sex for intelligence [38]. For all subsequent analyses, we considered the IQ scores for males and females together.

**Table 3:** Means, standard deviations, and analysis of variance by sex and zygosity for IQ.

	All		MZ		DZ		Female		Male		ANOVA			
*Age*	*M*	*SD*	*M*	*SD*	*M*	*SD*	*M*	*SD*	*M*	*SD*	*zyg*	*sex*	zyg*sex	*R^2^*
***2***	16.77	6.84	16.24	6.93	17.04	6.77	17.87	6.76	15.63	6.73	<0.01	<0.01	0.01	0.03
***3***	20.05	6.75	19.62	7.05	20.30	6.57	20.88	6.49	19.18	6.91	<0.01	<0.01	0.56	0.02
***4***	11.20	2.51	10.98	2.58	11.32	2.47	11.34	2.43	11.05	2.60	<0.01	<0.01	0.56	0.01
***7***	9.63	2.23	9.49	2.20	9.71	2.25	9.66	2.21	9.60	2.26	<0.01	0.32	0.63	<0.01
***9***	18.31	3.50	18.14	3.50	18.42	3.49	18.26	3.50	18.37	3.49	0.03	0.46	0.22	<0.01
***10***	28.30	5.56	27.98	5.63	28.49	5.51	28.00	5.53	28.68	5.58	0.02	<0.01	0.67	0.01
***12***	22.84	4.16	22.56	4.15	23.00	4.16	22.65	4.17	23.08	4.14	<0.01	<0.01	0.15	0.01
***14***	27.26	4.05	27.10	4.01	27.36	4.08	27.30	4.02	27.21	4.10	0.11	0.53	0.16	<0.01

MZ = monozygotic; DZ = dizygotic; M = mean; SD = standard deviation; ANOVA = analysis of variance; zyg/sex/zyg*sex = p-value associated variance attributable to zygosity/sex/the zygosity*sex interaction; R^2^ = variance explained by the ANOVA model.

Because similarity due to age and sex can contribute to phenotypic similarity and inflate estimates of C, as is standard practice in twin analyses [51], all verbal and nonverbal scales were corrected for the effects of age and sex before conducting twin analyses. Correlations between IQ measured at each age are presented in Table 2.

Below, results are presented for continuous moderation analyses of IQ moderated by three indices of SES: *SES index 1*, Parental education and occupation acquired at first contact (age 18 months); *SES index 2*, Parental education and occupation at age 7; and *SES index 3*, Parental income at age 9. At the end of this section, we present results for a discontinuous analysis, i.e., IQ as a function of stratified SES.

### SES index 1: Parental education and occupation at contact (age 18 months)

**Figure 2:**
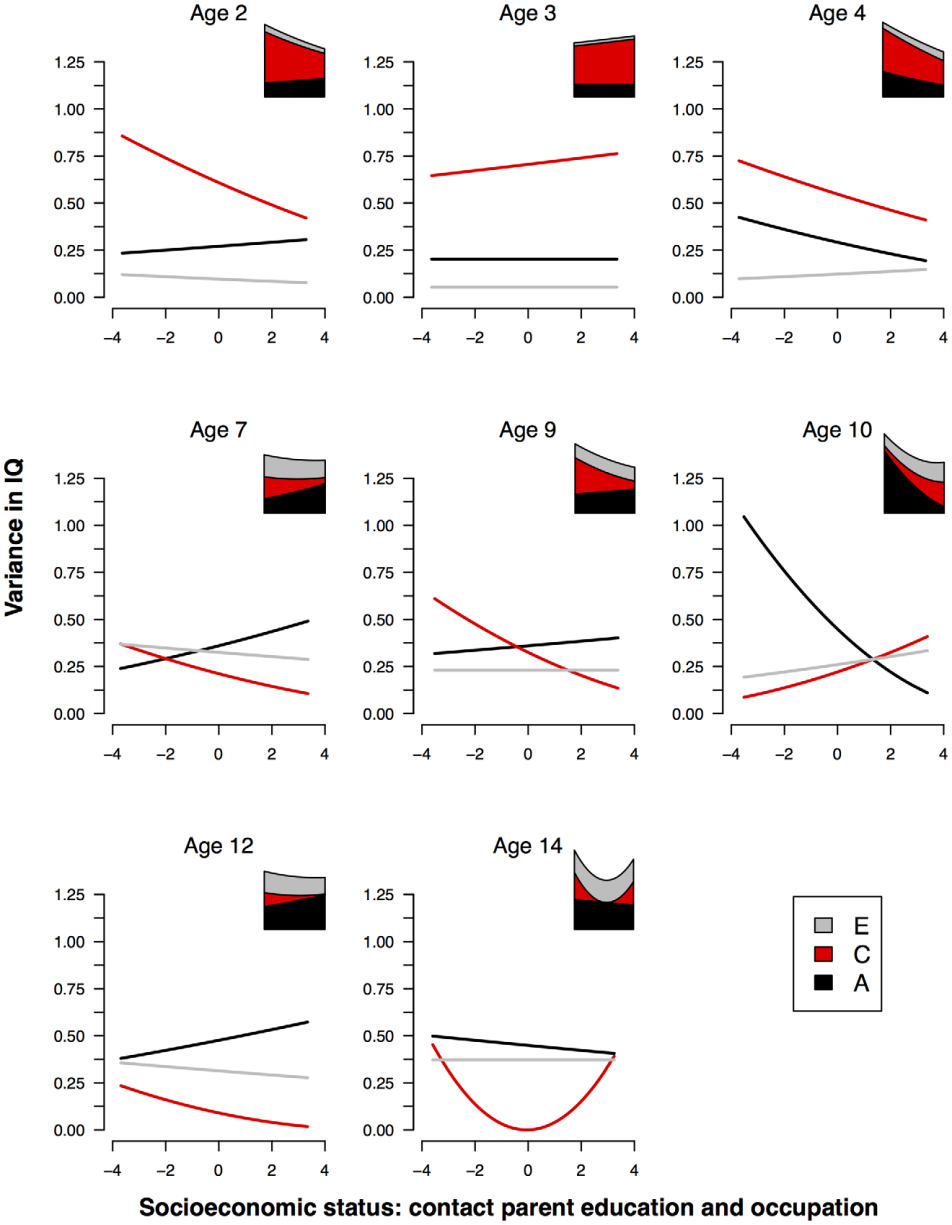
Unstandardized IQ variance components by SES index 1. Unstandardized genetic and environmental variance components for IQ as a function of first contact parental education and occupation (SES index 1). To the top right of each graph is a stacked plot showing the total variance in IQ as a function of SES.

Phenotypic correlations between SES (a unit-weighted composite of parental education and occupation acquired at contact) and IQ are presented in Table 4. From infancy to adolescence we found an increasing correlation between SES and IQ, from .08 to .37, as expected from the literature. A graphical summary of the continuous moderation analyses is presented in Figure 2. This visual summary of the SES moderation of IQ across the eight ages suggests three conclusions. First, the total variation in IQ changed with SES level: at ages 2, 4, 9, and 10 we found greater variance in low-SES families; at ages 3, 7 and 12 only small differences; and at age 14, greater variance at both ends of the SES distribution than around the mean.

**Table 4:** Phenotypic correlations between SES and IQ.

Age	Phenotypic correlation	N
	***SES index 1: parent education and occupation at 18 months***	
***2***	0.08 (0.05–0.11)	5110
***3***	0.17 (0.14–0.20)	4657
***4***	0.18 (0.16–0.20)	6726
***7***	0.32 (0.30–0.35)	4703
***9***	0.31 (0.27–0.34)	2966
***10***	0.26 (0.22–0.30)	2419
***12***	0.33 (0.30–0.35)	3972
***14***	0.37 (0.34–0.40)	2592
	***SES index 2: Parent education and occupation at age 7***	
***7***	0.29 (0.26–0.32)	4512
***9***	0.25 (0.22–0.29)	2610
***10***	0.22 (0.18–0.26)	2069
***12***	0.31 (0.28–0.34)	3588
***14***	0.33 (0.29–0.36)	2294
	***SES index 3: Family income at age 9***	
***9***	0.23 (0.20–0.26)	2959
***10***	0.17 (0.13–0.21)	2097
***12***	0.23 (0.19–0.27)	1822
***14***	0.26 (0.21–0.31)	1339

N = number of pair-wise observations (based on one randomly selected member from each twin pair); 95% confidence intervals shown in parentheses. All correlations significant at p<.001.

Second, except for a large drop in the A contribution with increasing SES at age 10, we found no substantial change in A across the eight ages: little or no change at ages 2, 3, 9, and 14, and small increases with increasing SES at ages 7 and 12. This suggests no consistent GxE interaction. Moreover, it should be noted that the only substantial GxE interaction at age 10 is in the opposite direction from that suggested in the literature: heritability is *greater* in low-SES families.

Third, differences in C were somewhat more consistent: at ages 2, 4, 7, 9, and 12, there was a drop in C with increasing SES. This suggests the presence of greater C in low-SES families.

Intra-class correlations (coefficients of twin similarity; [52]) are presented in Table 5. Doubling the differences between the MZ and DZ correlations provides a rough estimate of the heritability of IQ. These estimates show the expected pattern of increasing heritability with age, from 30% at age 2 to 46% at age 14. The extent to which MZ correlations are not explained by heritability provides an estimate of shared environment. These estimates show the expected pattern of decreasing shared environmental influence with age, from 61% at age 2 to 14% at age 14.

**Table 5:** Intra-class correlations (coefficients of twin similarity) for IQ by zygosity for twins with SES.

Age	ICC (95% CI)		N	
	*MZ*	*DZ*	*MZ*	*DZ*
***SES index 1***				
***2***	0.91 (0.90–0.92)	0.76 (0.75–0.77)	1677	3315
***3***	0.95 (0.95–0.96)	0.84 (0.82–0.85)	1200	2374
***4***	0.89 (0.88–0.90)	0.71 (0.69–0.73)	1238	2460
***7***	0.68 (0.65–0.71)	0.49 (0.46–0.52)	1264	2284
***9***	0.75 (0.72–0.78)	0.58 (0.54–0.61)	863	1495
***10***	0.73 (0.69–0.76)	0.50 (0.45–0.54)	685	1197
***12***	0.66 (0.62–0.70)	0.42 (0.37–0.47)	777	1242
***14***	0.60 (0.54–0.65)	0.37 (0.31–0.43)	563	894
***SES index 2***				
***7***	0.66 (0.64–0.69)	0.49 (0.46–0.52)	1614	2851
***9***	0.75 (0.72–0.77)	0.57 (0.53–0.60)	964	1595
***10***	0.74 (0.70–0.77)	0.50 (0.45–0.54)	744	1281
***12***	0.66 (0.62–0.69)	0.43 (0.39–0.46)	1310	2133
***14***	0.60 (0.55–0.64)	0.35 (0.30–0.40)	812	1201
***SES index 3***				
***9***	0.76 (0.73–0.78)	0.58 (0.55–0.61)	1084	1816
***10***	0.73 (0.69–0.76)	0.49 (0.44–0.53)	773	1285
***12***	0.65 (0.60–0.69)	0.42 (0.37–0.46)	685	1072
***14***	0.61 (0.55–0.66)	0.33 (0.27–0.40)	510	712

ICC (95% CI) = intra-class correlation coefficient (95% confidence interval); MZ = monozygotic; DZ = dizygotic; N = number of complete cases, i.e. number of pairs in which both twins have IQ data. NB. The formal estimation of variance components, using full information maximum likelihood structural equation modelling, included data from incomplete cases.

Table 6 shows the parameter estimates at each age derived from the full GxE interaction model with full information maximum likelihood estimation. Squaring the path estimate and dividing by the sum of the squared paths gives the standardized variance component: e.g., heritability or h^2^ = (a+*β_A_*M)^2^/((a+*β_A_*M)^2^+(c+*β_C_*M)^2^+(e+*β_E_*M)^2^). (A formal test of the significance of each moderated term in the interaction model, at each age, is shown in Table S1.)

**Table 6:** Genetic and environmental parameter estimates for IQ moderated by SES - full continuous moderator model.

	*Parameters*	*Age*							
		*2*	*3*	*4*	*7*	*9*	*10*	*12*	*14*
***SES index 1***	*a*	0.52	0.45	0.54	0.60	0.60	0.67	0.69	0.67
	*c*	0.78	0.84	0.74	0.46	0.57	0.47	0.30	0.01
	*e*	0.31	0.23	0.35	0.57	0.48	0.51	0.56	0.61
SES index 1 moderation of the	***β_A_***	0.01	0.00	−0.03	0.03	0.01	−0.10	0.02	−0.01
genetic and environmental	***β_C_***	−0.04	0.01	−0.03	−0.04	−0.06	0.05	−0.05	0.19
components of IQ	***β_E_***	−0.01	0.00	0.01	−0.01	0.00	0.02	−0.01	0.00
	*β_M_*	0.09	0.17	0.17	0.31	0.29	0.24	0.32	0.37
***SES index 2***	*a*				0.59	0.56	0.66	0.71	0.66
	*c*				0.47	0.60	0.49	0.24	0.17
	*e*				0.57	0.49	0.50	0.56	0.62
SES index 2 moderation of the	***β_A_***				0.04	0.01	−0.01	0.01	−0.07
genetic and environmental	***β_C_***				−0.06	−0.06	−0.05	−0.09	0.13
components of IQ	***β_E_***				0.00	0.00	0.00	−0.01	0.02
	*β_M_*				0.28	0.24	0.21	0.31	0.32
***SES index 3***	*a*					0.59	0.66	0.76	0.70
	*c*					0.60	0.50	0.16	0.20
	*e*					0.48	0.51	0.55	0.61
SES index 3 moderation of the	***β_A_***					−0.01	−0.04	−0.02	0.05
genetic and environmental	***β_C_***					−0.05	−0.04	−0.16	−0.16
components of IQ	***β_E_***					0.00	0.01	0.00	−0.03
	*β_M_*					0.23	0.17	0.25	0.29

At ages 3, 7, and 12 the best-fitting model, as indicated by AIC, was one with no moderation of either genetic or environmental components. At age 2, the best fitting model, as indicated by AIC, was one with no genetic moderation. The p-value showing model fit for individually dropped parameters suggests only moderation of the C term is significant (*β_C_* = −.04). At age 4, the best-fitting model was one with moderation of both A and C terms (*β_A_* = −.03, *β_C_* = −.03). At age 9, moderation of only the C term was significant (*β_C_* = −.06). Age 10 showed a significant decrease in A with increasing SES (*β_A_* = −.10). At age 14, the best fitting model, as indicated by AIC, suggested significant moderation of the C term (*β_C_* = −.19).

All significant genetic and environmental moderation was in the direction of greater variance in IQ explained at lower levels of SES.

### SES index 2: Parental education and occupation at age 7

Phenotypic correlations between SES (a unit-weighted composite of parental education and occupation assessed at age 7) and IQ show a similar pattern of increasing correlation with age, and are in the range 0.22–0.33 (Table 4). Intra-class correlations for twins with data on 7-year parental education and occupation are presented in Table 5. Rough estimates of variance components calculated by doubling the differences between the MZ and DZ correlations are similar to estimates for twins with SES index 1 data.

**Figure 3:**
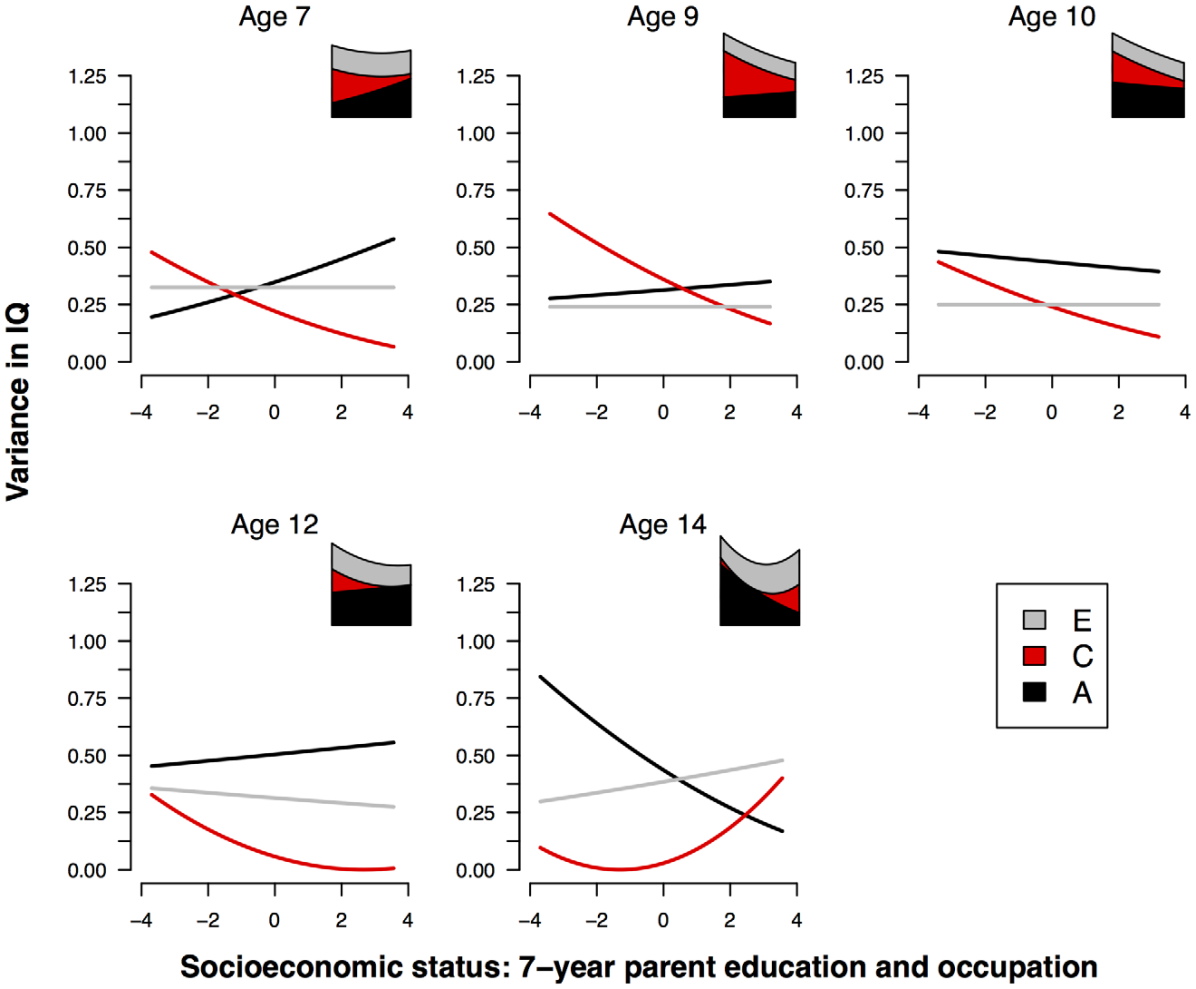
Unstandardized IQ variance components by SES index 2. Unstandardized genetic and environmental variance components for IQ as a function of 7-year parental education and occupation (SES index 2). To the top right of each graph is a stacked plot showing the total variance in IQ as a function of SES.

A graphical summary of the continuous moderation analyses is presented in Figure 3. Inspection of the visual summary of the interaction analyses reveals a consistent increase in the effect of the shared environment on IQ with decreasing SES, coupled with an increase in the variance in IQ in low-SES families - most notably at ages 9, 10 and 12.

Table 6 shows the parameter estimates at each age derived from the full GxE interaction model with full information maximum likelihood estimation. (A formal test of the significance of each moderated term in the interaction model, at each age, is shown in Table S2.)

At ages 7 and 14, the best fitting model as indicated by AIC was one with no moderation of genetic or environmental components of intelligence. At all other ages (9, 10, and 12) the best fitting model included only moderation of the C component (*β_C_* = −.06, *β_C_* = −.05, and *β_C_* = −.09 respectively).

### SES index 3: Parental income at age 9

Phenotypic correlations between SES (family income at age 9) and IQ are presented in Table 4. As for SES indices 1 and 2, we find a pattern of increasing correlation between IQ and SES index 3 with age, with correlations in the range 0.17–0.26. Intra-class correlations by zygosity for twins with 9-year family income data are presented in Table 5. Again, rough estimates of variance components found by doubling the differences between the MZ and DZ correlations are similar to estimates for twins with SES index 1 and 2 data.

**Figure 4:**
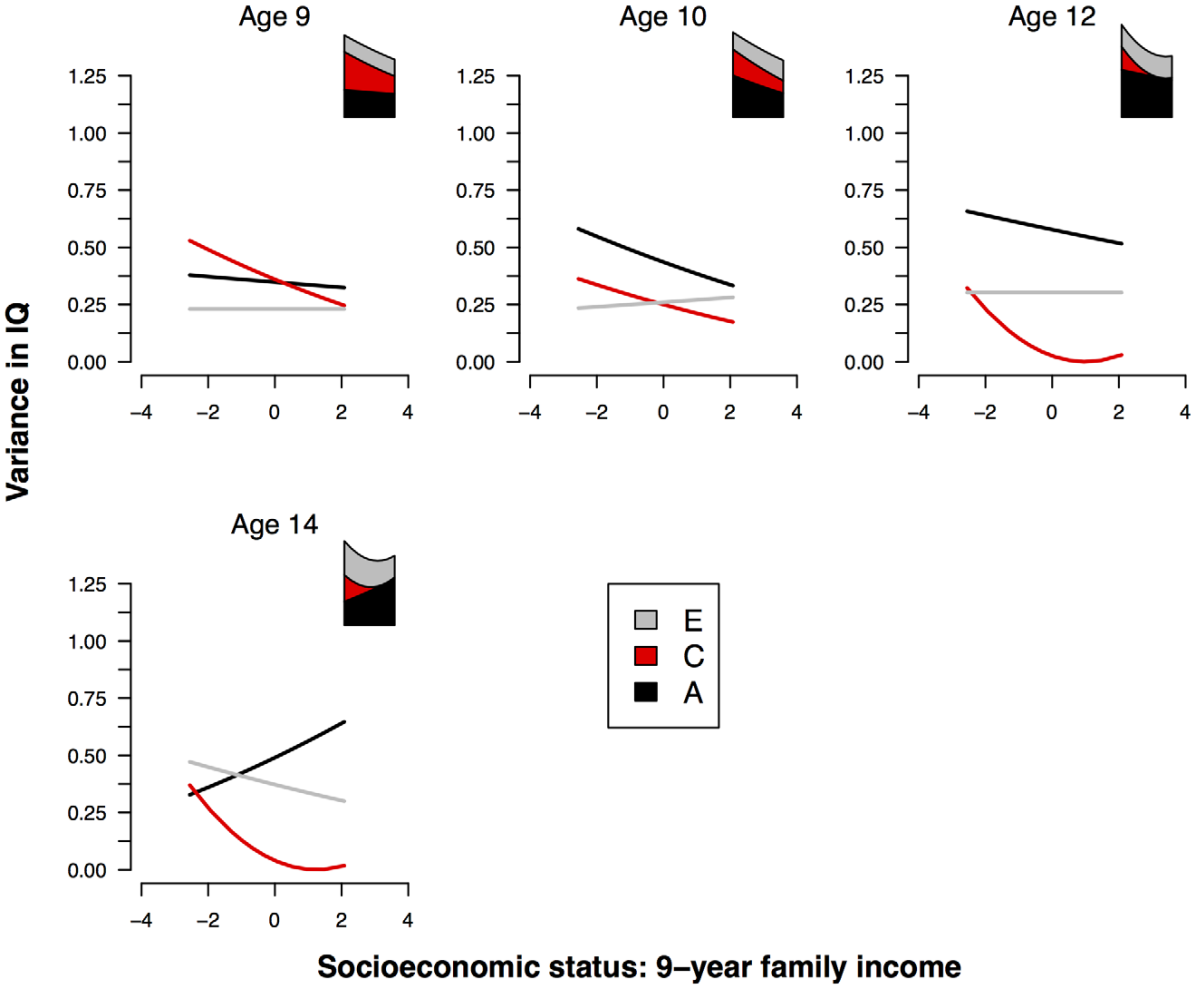
Unstandardized IQ variance components by SES index 3. Unstandardized genetic and environmental variance components for IQ as a function of 9-year family income (SES index 3). To the top right of each graph is a stacked plot showing the total variance in IQ as a function of SES.

A graphical summary of the continuous moderation analyses at ages 9, 10, 12 and 14 is presented in Figure 4. As for the other indices of SES, the visual summary of the interaction analyses reveals an increase in the variance in IQ in low-SES families, an increase in the effect of the shared environment on IQ with decreasing SES, and inconsistent differences in genetic effect.

Table 6 shows the parameter estimates at each age derived from the full GxE interaction model with full information maximum likelihood estimation. (A formal test of the significance of each moderated term in the interaction model, at each age, is shown in Table S3.)

At all ages (9, 10, 12, and 14), the best fitting model as indicated by AIC includes (in addition to the main effect of SES) only moderation of the shared environmental component (*β_C_* = −.05, *β_C_* = −.04, *β_C_* = −.16, and *β_C_* = −.16 respectively).

### What is the most parsimonious account of the moderating effect of SES?

Summarized in Table 7 are the best-fitting models at each age, for each of the three indices of SES. An asterisk indicates the best-fitting model (as indicated by AIC). It should be noted that at each age, in testing the significance of each parameter in the model, AIC suggests very little difference between each of the accounts of the data (see last column in Tables S1, S2, and S3). Accepting this small difference, three results are worth highlighting. First, the only significant GxE interaction with SES index 1 found for *g* at age 10 (higher heritability in low-SES families) disappears with the more proximal measures of SES at ages 7 and 9. Second, the best fitting model indicates no interaction of any kind at three ages for SES index 1 (ages 3, 7, and 12), and for two ages for SES index 2 (ages 7 and 14). Third, moderation of the shared environmental component of *g* is indicated at four of eight ages for SES index 1, three of five ages for SES index 2, and four of four ages for SES index 3. Thus, the most consistent result across ages and across the three indices of SES is moderation of the influence of shared environment on children's intelligence - an *environment*-environment interaction.

**Table 7:** Summary of best fitting model (as indicated by AIC) for three indices of SES.

§ *Best fitting model*	*SES index 1*								*SES index 2*					*SES index 3*			
	*2*	*3*	*4*	*7*	*9*	*10*	*12*	*14*	*7*	*9*	*10*	*12*	*14*	*9*	*10*	*12*	*14*
*ace β_A_ β_C_ β_E_ β_M_*																	
*β_A_ = 0*	◯																
*β_C_ = 0*																	
*β_E_ = 0*			◯														
*β_A_ = β_C_ = 0*																	
*β_A_ = β_E_ = 0*								⊙		⊙	⊙	⊙		⊙	⊙	⊙	⊙
*β_C_ = β_E_ = 0*						•											
*β_A_ = β_C_ = β_E_ = 0*		•		•			•		•				•				

§ Best fitting model as indicated by Akaike's information criterion (AIC); SES index 1 = a composite of parental education and occupation acquired when the TEDS twins were 18 months old; SES index 2 = a composite of parental education and occupation acquired when the TEDS twins were 7 years old; SES index 3 = family income measured when the TEDS twins were 9 years old; a, c, e = unmoderated genetic, shared, and nonshared environmental path coefficients; β_A_, β_C_, β_E_ = moderated genetic, shared, and nonshared environmental path coefficients; β_M_ = main effect of moderator on mean of IQ; ◯ = includes C moderation; ⊙ = C moderation only.

### Performance of the continuous moderator model with simulated data

**Figure 5:**
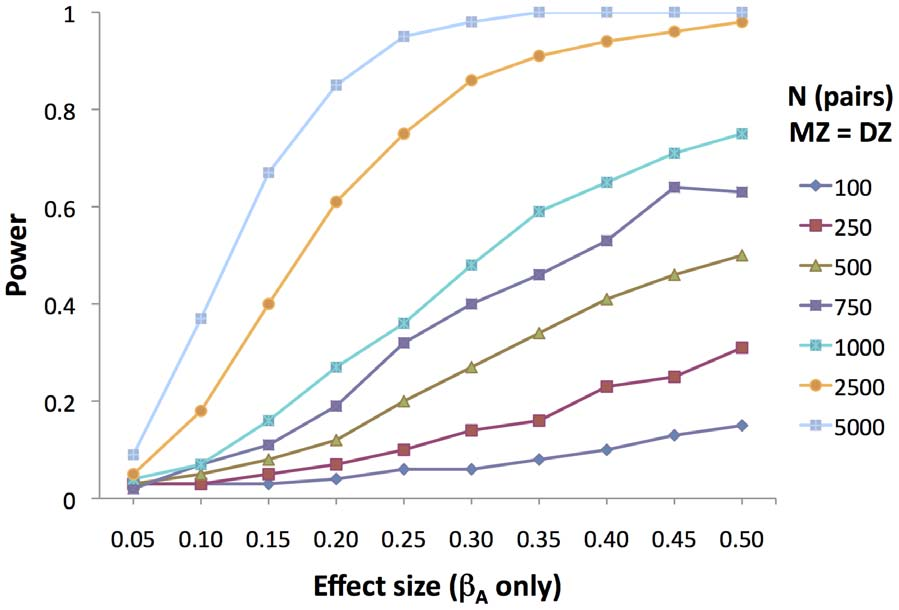
Power to detect GxE when only genetic moderation is simulated. Power to detect the presence of a genetic moderation with the continuous moderator model (genetic moderation only simulated). Equal number of MZ and DZ twin pairs simulated (N = 500, means 500 MZ and 500 DZ pairs). N = sample size; MZ = monozygotic; DZ = dizygotic; β_A_ = moderated element of genetic path coefficient.

In order to estimate power of the continuous model to detect genetic moderation under conditions of genetic moderation only, we set parameters as follows; *a* = *c* = *e* = 1; *β_C_* = *β_E_* = 0. We simulated a range of genetic moderation (*β_A_*) between 0.05 and 0.50. We generated 1000 replicates for a range of sample sizes, with equal numbers of MZ and DZ twin pairs. Figure 5 shows that a sample size of about 2500 pairs of MZ and DZ twins each is needed to detect an effect size (genetic moderation) of between 0.25 and 0.30 with 80% power. A genetic moderation of 0.25 translates to a difference in heritability of about 11% at −2SD of the moderator to about 53% at +2SD of the moderator (at the simulated parameter values).

**Figure 6:**
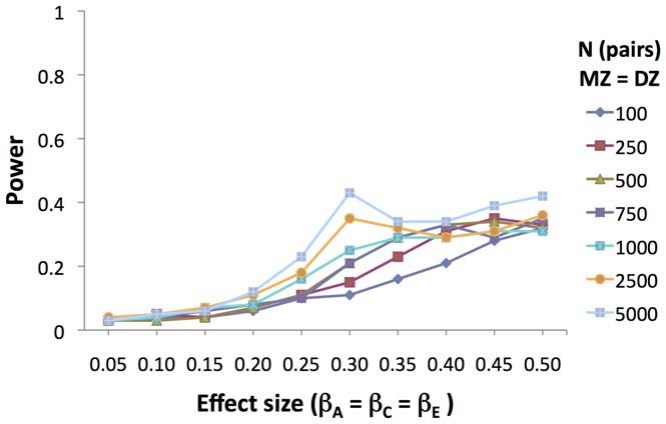
Power to detect GxE when genetic and environmental moderation are simulated. Power to detect the presence of a genetic moderation with the continuous moderator model (equal genetic, shared and nonshared environmental moderation simulated). Equal number of MZ and DZ twin pairs simulated (N = 500, means 500 MZ and 500 DZ pairs). N = sample size; MZ = monozygotic; DZ = dizygotic; β_A_, β_C_, β_E_ = moderated elements of genetic, shared environmental, and nonshared environmental path coefficients.

Second, to explore how the model performed when moderation of all three terms is present, we simulated data with parameters set as follows: *a* = *c* = *e* = 1; and, *β_C_* = *β_E_* = *β_A_* = a range of values between 0.05 and 0.50. Again, we generated 1000 replicates for each sample and effect size, and estimated the model's ability to detect the presence of the genetic moderation only, i.e. a 1df test. Figure 6 is more informative about model performance than power per se. With equal moderation of the genetic, shared, and nonshared environmental components, above a moderation of 0.30 (moderated coefficient 30% of the unmoderated coefficient, i.e., *β_A_* = 0.30*a) the model does not perform well when assessing the significance of just the genetic moderation (a 1df test). Purcell's [18] simulations suggest that this would also be the case when testing only moderation of the shared environment.

The simulations summarized in Figures 3a and 3b perhaps illustrate the best and worst case scenario for the continuous moderator model. In the case of genetic moderation only, the model performs well, and increasing sample size increases power to detect genetic moderation. However, as noted by Purcell [18], the model does not do well at distinguishing between genetic and shared environmental moderation when both are present, and one proceeds by testing one term at a time.

### Discontinuous analysis of low-SES versus high-SES groups

Because several studies explored GxE interaction by comparing ACE estimates, or twin correlations, in low-and high-SES groups (see Table 1), we compared results of our continuous moderator analysis with the results for a discontinuous analysis. We estimated variance components in low- and high-SES groups, and tested whether these could be equated – a *heterogeneity* analysis. Although these discontinuous analyses have usually ignored variance differences between groups by using twin correlations (which standardize variances between groups), heterogeneity analysis provided components of raw variance which we present along with the standardized estimates to highlight the difference between components of raw and standardized variance.

**Figure 7:**
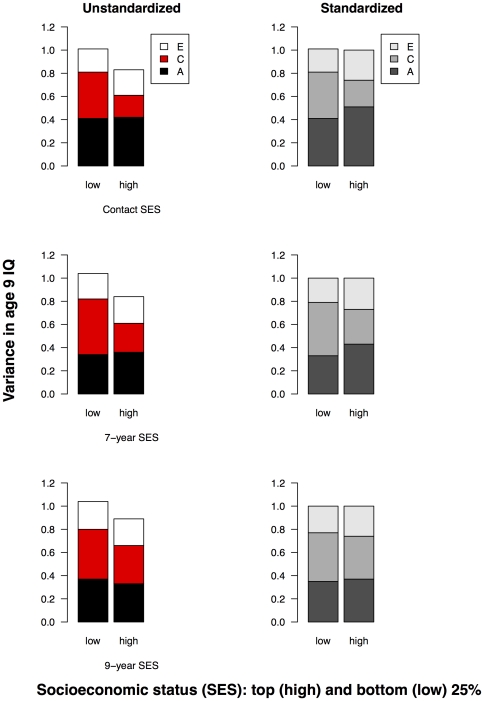
Age 9 IQ in low- and high-SES groups – heterogeneity analysis. Variance components of 9-year IQ in low- and high-SES families (bottom and top 25% of SES distribution). Top, middle, and bottom rows show IQ as a function of 18-month, 7-year, and 9-year SES respectively (SES indices 1, 2, and 3). In the left column are the unstandardized estimates; in the right column are the standardized estimates.

We present results for age 9 IQ, which showed the most consistent C interaction across the three SES indices. We split the sample into quartiles and compared the variance components derived for the top and bottom 25% of the SES distribution. In Figure 7, rows 1, 2, and 3 show age 9 IQ components as a function of SES indices 1, 2, and 3 respectively; in the left column are the components of raw variance, in the right hand column are the standardized estimates. The unstandardized estimates show greater total variance for the low-SES groups and this excess variance can be attributed to greater shared environment for the low-SES group. Shared environment is significantly greater in the low-SES group for SES indices 1 (low-SES C = .40 {95% confidence interval (CI) = .27–.53}; high-SES C = .19 {95% CI = .08–.31}) and 2 (low-SES C = .48 {.34–.62}; high-SES C = .25 {.13–.37}). Equating C in low- and high-SES groups significantly reduced model fit (SES index 1: Δ−2lnL = 5.45, Δdf = 1, ΔAIC = 3.45, p = .02; SES index 2: Δ−2lnL = 3.572, Δdf = 1, ΔAIC = 5.57, p = .02). In contrast, heritability estimates are identical for the low- and high-SES groups. The standardized estimates also show greater C in the low-SES group for SES indices 1 and 2; however, standardizing the variance components in the two groups artificially increases estimates of A in the high-SES group.

In summary, this discontinuous analysis of low-SES versus high-SES groups generally confirms the results of our continuous moderator analysis for the largest interaction effect, despite a great loss in power for the discontinuous analysis [18].

## Discussion

We attempted to replicate the finding that parental SES moderates the heritability of children's intelligence, with a greater genetic contribution to IQ in high-SES families compared to low-SES families. In a large UK-representative sample, we did not find evidence for the presence of such a *gene*-environment interaction across childhood and adolescence. At only one of the eight ages, age 10, did we find a significant moderation of the genetic contribution to IQ. However, the GxE interaction was in the opposite direction from that predicted by the environmental disadvantage hypothesis, and moreover, was not significant with a more proximal measure of parental education and occupation. Instead, using three different indices of SES, at eight ages from infancy through adolescence the emerging pattern appears to be one of *environment*-environment interaction rather than *gene*-environment interaction: shared experiences explain more of the variance in children's performance on IQ tests in more disadvantaged backgrounds.

### Environmental moderation of shared experiences

How can the present finding of SES moderation of the shared environmental effect on IQ, be reconciled to the reports of SES moderation of the genetic component of IQ? An increase in the contribution of C in lower-SES families would seem to require a reduction in the relative contribution of A because environmental and genetic variance components are complementary, and explain 100% of the variance. However, this is only the case for standardized components that are forced to sum to 100% regardless of total variance differences. Our most consistent finding is that total IQ variance is greater in lower-SES families, which must be caused by greater A, C, or E components of variance in lower-SES families. Although the power demands are daunting to disentangle A and C sources of this increased variance in lower-SES families, data from our large sample suggests that the source is C rather than A. The genetic effect does not differ for low- and high-SES groups using unstandardized estimates (A, C, and E) that take into account the greater total variance in the low-SES group, but the *relative* contribution of genes – heritability or h^2^ = A/(A+C+E)) – is lower in low-SES families because the shared environmental effect increases.

Children from low-SES families face many physical and psychosocial environmental handicaps for their cognitive development [53]. For example, low-SES children are read to less, have fewer books, less access to computers, and tend to watch more television. Parents tend to be less responsive to children in low-SES families, participate less in their children's school activities, and are more authoritarian. Children from more disadvantaged backgrounds tend to experience more instability, come from noisier, more crowded homes, and live in disadvantaged neighbourhoods with poorer facilities and inferior schools (for a recent review of the correlates of low-SES see [53]). To the extent that children growing up together experience these environments similarly, their cumulative effects are captured by the C component in a twin model; experiences such as these seem likely to contribute to the observed greater variation in the cognitive ability performance of children from low-SES families.

### Sampling, age differences, and power to detect C

What factors contribute to the inconsistency in the literature (Table 1)? We suggest three possibilities: sampling, age range, and power to distinguish moderation by A and C. First, a general concern is that sampling from different ranges of a putative moderator distribution (low, medium, or high levels), can lead to different conclusions about the presence or absence of a GxE interaction [54]. Factors that are additive across the entire range of a moderator may appear to be interacting within small windows at the extremes of a dose-response curve [3]. However, it is also possible a different gene-environment dynamic exists at the extremes of SES [12]. Children from average- and high-SES families receive adequate educational resources, parent-child interaction, and orderly homes within safe neighbourhoods. However, below a certain threshold of environmental quality, children's experience could begin to have a negative impact on their cognitive ability. For example, the National Collaborative Perinatal Project oversampled families from an extremely impoverished background, with a quarter of the families on incomes below the poverty line [10]. Extreme levels of the environment, however, cannot be the sole reason for the inconsistent reports; the same team replicated the GxE interaction found in the National Collaborative Perinatal Project [10], in a sample representative of the US population [9].

Differences between countries is another possible sampling issue for two overlapping reasons: the relationship of the SES measures to each other, and their relationship to IQ. First, the traditional measures of SES – family income, parental education, and occupational status [55] – may differ in relation to each other by population group [56] and may also depend on country-specific political and historical background [57]. The extent to which income, education, and occupation successfully capture financial, human, and social capital and their effect on child development are discussed thoroughly elsewhere [55]. In the present study, we combined education and occupation to better capture a broader construct of SES, and benefitted from being able to compare the measure at two ages; we treated income separately as we only had this measure from age 9 on.

Second, the magnitude and nature of the effect of SES on children's IQ could differ in different countries [57], such as the UK versus the US. Although this possibility has not been systematically tested, inspection of the studies in Table 1 is consistent with the hypothesis of differences between European and US samples. Within the European studies, only one reported an increasing heritability of IQ with SES [7]; this finding was based on estimates of twin correlations from a small sample. Among the US samples, with the exception of inconclusive results in a study with very small sample size [14], the only non-replication of the greater heritability with increasing SES finding was in an older sample, with an age range of 16 to 30 years [15].

We believe that sample age is a particularly important factor in the inconsistent findings. Because heritability increases and shared environmental influence decreases from childhood to adulthood [21], [22], developmental differences in moderation could be expected. Two of the four studies in Table 1 that do not find greater heritability of IQ in higher SES are in older samples, ranging in age from 16 to 49 years [15], [16]. The third non-replication was based on a small sample and unreliable estimates [14]. The last of the four non-replications involved an earlier analysis in the TEDS sample. This earlier analysis found no significant moderation of the heritability of age 4 IQ by SES, but did find moderation of the genetic effect by family chaos and parent-child communication [17]. Using the continuous moderator model, the present study suggests that SES does in fact moderate the relative contributions of A and C to variance in age 4 IQ – we suggest this is driven by a moderation of C.

Detecting modest shared environmental effects in the presence of larger genetic and nonshared environmental effects requires large twin samples [58]. This difficulty is compounded by the fact that the shared environmental contribution to general cognitive ability diminishes with age. We suggest moderation of the shared environmental effect on IQ could go undetected in smaller samples and that it could be misinterpreted as genetic moderation given the low power of the continuous moderator model to distinguish between moderation of the genetic and shared environmental variance components. Even with a relatively large sample, as in the present study, comparing the fit of nested models yields little difference in their ability to explain the data, as indicated by the small AIC differences at every age and for every SES index (Tables S1, S2, and S3).

Several quantitative genetic approaches have been used to investigate moderation of the genetic effect on IQ. These include the heterogeneity model (e.g., splitting the sample into groups “low” versus “high” on the moderator), regression models with an interaction term (e.g. extended DF regression), and the continuous moderator model. The continuous moderator model is the most powerful approach, allowing the use of full information maximum likelihood to estimate potential moderation of latent variance components while simultaneously controlling for the confounding effects of gene-environment correlation. Because the interactions tested by the various approaches are *statistical* in nature, they are necessarily dependent on measurement scale, analytical model, and the assumptions underlying the model. Establishing a mechanism for moderation of the effect of genes, such as a change in gene expression, is several steps removed from finding moderation as a latent genetic population variance component [3]. Likewise, statistical moderation of a shared environmental component needs to be experimentally investigated to understand the real-world mechanisms behind the moderation.

### Conclusion

The notion that heritability may be lower in lower-SES families is appealing, in part because of its environmental implications: If heritability is lower in lower-SES families, it suggests that environmental interventions might be more effective in boosting cognitive development for children in lower-SES families. The present study, which is based on a large UK-representative sample of children followed longitudinally, leads to a similar implication. Although the genetic influence on IQ is the same in lower-SES families, shared environmental influence appears to be greater in lower-SES families, suggesting that family-based environmental interventions might be more effective in these families. However, two further aspects of the results temper the policy implications of this finding. First, shared environmental influence is found in both lower- and higher-SES families and the difference in shared environmental influence between them is modest. Second, shared environmental influences on IQ decline from childhood to adulthood so that these influences might not have an impact in the long run.

## Supporting Information

Table S1 **Continuous moderator model fit – SES index 1.** Model fit for twins with parental education and occupation at 18 months. Bold rows show best fitting model as indicated by AIC.
(DOC)

Table S2 **Continuous moderator model fit – SES index 2.** Model fit for twins with 7-year parental education and occupation. Bold rows show best fitting model as indicated by AIC.
(DOC)

Table S3 **Continous moderator model fit – SES index 3.** Model fit for twins with 9-year family income at age 9. Bold rows show best fitting model as indicated by AIC.
(DOC)
