# Supplementary material for: Socioeconomic Status (SES) and Children's Intelligence (IQ): In a UK-Representative Sample SES Moderates the Environmental, Not Genetic, Effect on IQ
Source: PLoS One. 2012 Feb 1;7(2):e30320. doi: 10.1371/journal.pone.0030320 (PMC3270016; doi:10.1371/journal.pone.0030320)
Supplement: Table S1 — Continuous moderator model fit – SES index 1. Model fit for twins with parental education and occupation at 18 months. Bold rows show best fitting model as indicated by AIC. (DOC) [file pone.0030320.s001.doc]

**Table S1** Continuous moderator model fit for IQ by SES index 1 – parental education and occupation at 18 months.

| ***Age*** | ***Model*** | ***Δ-2lnL*** | ***Δdf*** | ***p-value*** | ***AIC*** |
| --- | --- | --- | --- | --- | --- |
| ***2*** | *ace β_A_ β_C_ β_E_ β_M_* | -- | -- | -- | 2667.258 |
|  | **β_A_ = 0** | **1.298** | **1** | **0.254** | **2666.556** |
|  | β_C_ = 0 | 10.493 | 1 | *0.001 | 2675.752 |
|  | β_E_ = 0 | 3.330 | 1 | 0.068 | 2668.588 |
|  | β_A_ = β_C_ = 0 | 10.768 | 2 | *0.005 | 2674.027 |
|  | β_A_ = β_E_ = 0 | 3.378 | 2 | 0.184 | 2666.637 |
|  | β_C_ = β_E_ = 0 | 11.992 | 2 | *0.002 | 2675.250 |
|  | β_A_ = β_C_ = β_E_ = 0 | 13.743 | 3 | *0.003 | 2675.001 |
|  |  |  |  |  |  |
| ***3*** | *ace β_A_ β_C_ β_E_ β_M_* | -- | -- | -- | 795.956 |
|  | β_A_ = 0 | 0.010 | 1 | 0.921 | 793.966 |
|  | β_C_ = 0 | 0.216 | 1 | 0.641 | 794.172 |
|  | β_E_ = 0 | 0.210 | 1 | 0.646 | 794.167 |
|  | β_A_ = β_C_ = 0 | 0.225 | 2 | 0.894 | 792.181 |
|  | β_A_ = β_E_ = 0 | 0.223 | 2 | 0.894 | 792.180 |
|  | β_C_ = β_E_ = 0 | 0.381 | 2 | 0.827 | 792.337 |
|  | **β_A_ = β_C_ = β_E_ = 0** | **0.466** | **3** | **0.926** | **790.422** |
|  |  |  |  |  |  |
| ***4*** | *ace β_A_ β_C_ β_E_ β_M_* | -- | -- | -- | 4490.210 |
|  | β_A_ = 0 | 5.018 | 1 | *0.025 | 4493.228 |
|  | β_C_ = 0 | 4.946 | 1 | *0.026 | 4493.156 |
|  | **β_E_ = 0** | **1.312** | **1** | **0.252** | **4489.522** |
|  | β_A_ = β_C_ = 0 | 23.583 | 2 | *0.000 | 4509.793 |
|  | β_A_ = β_E_ = 0 | 5.068 | 2 | 0.079 | 4491.278 |
|  | β_C_ = β_E_ = 0 | 8.138 | 2 | *0.017 | 4494.347 |
|  | β_A_ = β_C_ = β_E_ = 0 | 24.109 | 3 | *0.000 | 4508.319 |
|  |  |  |  |  |  |
| ***7*** | *ace β_A_ β_C_ β_E_ β_M_* | -- | -- | -- | 5340.294 |
|  | β_A_ = 0 | 1.283 | 1 | 0.257 | 5339.577 |
|  | β_C_ = 0 | 1.432 | 1 | 0.231 | 5339.727 |
|  | β_E_ = 0 | 0.547 | 1 | 0.459 | 5338.841 |
|  | β_A_ = β_C_ = 0 | 1.459 | 2 | 0.482 | 5337.753 |
|  | β_A_ = β_E_ = 0 | 1.292 | 2 | 0.524 | 5337.586 |
|  | β_C_ = β_E_ = 0 | 1.448 | 2 | 0.484 | 5337.743 |
|  | **β_A_ = β_C_ = β_E_ = 0** | **1.460** | **3** | **0.692** | **5335.754** |
|  |  |  |  |  |  |
| ***9*** | *ace β_A_ β_C_ β_E_ β_M_* | -- | -- | -- | 2834.482 |
|  | β_A_ = 0 | 0.049 | 1 | 0.826 | 2832.530 |
|  | β_C_ = 0 | 5.255 | 1 | *0.022 | 2837.737 |
|  | β_E_ = 0 | 0.169 | 1 | 0.681 | 2832.651 |
|  | β_A_ = β_C_ = 0 | 11.526 | 2 | *0.003 | 2842.001 |
|  | **β_A_ = β_E_ = 0** | **0.536** | **2** | **0.765** | **2831.018** |
|  | β_C_ = β_E_ = 0 | 7.199 | 2 | *0.027 | 2837.682 |
|  | β_A_ = β_C_ = β_E_ = 0 | 11.529 | 3 | *0.009 | 2840.011 |
|  |  |  |  |  |  |
| ***10*** | *ace β_A_ β_C_ β_E_ β_M_* | -- | -- | -- | 2638.801 |
|  | β_A_ = 0 | 3.912 | 1 | *0.047 | 2640.713 |
|  | β_C_ = 0 | 1.086 | 1 | 0.297 | 2637.888 |
|  | β_E_ = 0 | 1.726 | 1 | 0.189 | 2638.528 |
|  | β_A_ = β_C_ = 0 | 10.489 | 2 | *0.005 | 2645.291 |
|  | β_A_ = β_E_ = 0 | 3.924 | 2 | 0.141 | 2638.725 |
|  | **β_C_ = β_E_ = 0** | **1.884** | **2** | **0.389** | **2636.686** |
|  | β_A_ = β_C_ = β_E_ = 0 | 11.329 | 3 | *0.010 | 2644.131 |
|  |  |  |  |  |  |
| ***12*** | *ace β_A_ β_C_ β_E_ β_M_* | -- | -- | -- | 4597.302 |
|  | β_A_ = 0 | 0.304 | 1 | 0.581 | 4595.607 |
|  | β_C_ = 0 | 0.316 | 1 | 0.574 | 4595.618 |
|  | β_E_ = 0 | 0.959 | 1 | 0.327 | 4596.261 |
|  | β_A_ = β_C_ = 0 | 0.317 | 2 | 0.853 | 4593.619 |
|  | β_A_ = β_E_ = 0 | 1.199 | 2 | 0.549 | 4594.501 |
|  | β_C_ = β_E_ = 0 | 1.054 | 2 | 0.590 | 4594.357 |
|  | **β_A_ = β_C_ = β_E_ = 0** | **1.435** | **3** | **0.697** | **4592.738** |
|  |  |  |  |  |  |
| ***14*** | *ace β_A_ β_C_ β_E_ β_M_* | -- | -- | -- | 3105.441 |
|  | β_A_ = 0 | 0.000 | 1 | 0.976 | 3103.442 |
|  | β_C_ = 0 | 6.471 | 1 | *0.011 | 3109.912 |
|  | β_E_ = 0 | 0.020 | 1 | 0.886 | 3103.462 |
|  | β_A_ = β_C_ = 0 | 6.539 | 2 | *0.038 | 3107.981 |
|  | **β_A_ = β_E_ = 0** | **0.035** | **2** | **0.983** | **3101.476** |
|  | β_C_ = β_E_ = 0 | 6.473 | 2 | *0.039 | 3107.914 |
|  | β_A_ = β_C_ = β_E_ = 0 | 6.744 | 3 | 0.084 | 3106.086 |

Model fit for twins with parental education and occupation at 18 months. Bold rows show best fitting model as indicated by AIC.

* = significantly worse model fit as indicated by p-value
