# Supplementary material for: Socioeconomic Status (SES) and Children's Intelligence (IQ): In a UK-Representative Sample SES Moderates the Environmental, Not Genetic, Effect on IQ
Source: PLoS One. 2012 Feb 1;7(2):e30320. doi: 10.1371/journal.pone.0030320 (PMC3270016; doi:10.1371/journal.pone.0030320)
Supplement: Table S2 — Continuous moderator model fit – SES index 2. Model fit for twins with 7-year parental education and occupation. Bold rows show best fitting model as indicated by AIC. (DOC) [file pone.0030320.s002.doc]

**Table S2** Continuous moderator model fit for IQ by SES index 2 - parental education and occupation at age 7

| ***Age*** | ***Model*** | ***Δ-2lnL*** | ***Δdf*** | ***p-value*** | ***AIC*** |
| --- | --- | --- | --- | --- | --- |
| ***7*** | *ace β_A_ β_C_ β_E_ β_M_* | -- | -- | -- | 5154.816 |
|  | *β_A_ = 0* | 1.545 | 1 | 0.214 | 5154.361 |
|  | *β_C_ = 0* | 2.512 | 1 | 0.113 | 5155.328 |
|  | *β_E_ = 0* | 0.094 | 1 | 0.760 | 5152.910 |
|  | *β_A_ = β_C_ = 0* | 2.762 | 2 | 0.251 | 5153.579 |
|  | *β_A_ = β_E_ = 0* | 2.409 | 2 | 0.299 | 5153.226 |
|  | *β_C_ = β_E_ = 0* | 3.126 | 2 | 0.209 | 5153.942 |
|  | ***β_A_ = β_C_ = β_E_ = 0*** | **3.126** | **3** | **0.373** | **5151.942** |
|  |  |  |  |  |  |
| ***9*** | *ace β_A_ β_C_ β_E_ β_M_* | -- | -- | -- | 2530.328 |
|  | *β_A_ = 0* | 0.185 | 1 | 0.667 | 2528.513 |
|  | *β_C_ = 0* | 4.656 | 1 | *0.031 | 2532.984 |
|  | *β_E_ = 0* | 0.023 | 1 | 0.879 | 2528.351 |
|  | *β_A_ = β_C_ = 0* | 9.504 | 2 | *0.009 | 2535.832 |
|  | ***β_A_ = β_E_ = 0*** | **0.210** | **2** | **0.900** | **2526.538** |
|  | *β_C_ = β_E_ = 0* | 5.267 | 2 | 0.072 | 2531.595 |
|  | *β_A_ = β_C_ = β_E_ = 0* | 9.834 | 3 | *0.020 | 2534.162 |
|  |  |  |  |  |  |
| ***10*** | *ace β_A_ β_C_ β_E_ β_M_* | -- | -- | -- | 2221.107 |
|  | *β_A_ = 0* | 0.128 | 1 | 0.720 | 2219.235 |
|  | *β_C_ = 0* | 1.279 | 1 | 0.258 | 2220.386 |
|  | *β_E_ = 0* | 0.122 | 1 | 0.727 | 2219.229 |
|  | *β_A_ = β_C_ = 0* | 6.166 | 2 | *0.046 | 2223.273 |
|  | ***β_A_ = β_E_ = 0*** | **0.476** | **2** | **0.788** | **2217.583** |
|  | *β_C_ = β_E_ = 0* | 1.281 | 2 | 0.527 | 2218.388 |
|  | *β_A_ = β_C_ = β_E_ = 0* | 7.650 | 3 | *0.054 | 2222.757 |
|  |  |  |  |  |  |
| ***12*** | *ace β_A_ β_C_ β_E_ β_M_* | -- | -- | -- | 4174.114 |
|  | *β_A_ = 0* | 4.280 | 1 | *0.039 | 4176.394 |
|  | *β_C_ = 0* | 0.826 | 1 | 0.364 | 4172.940 |
|  | *β_E_ = 0* | 0.891 | 1 | 0.345 | 4173.005 |
|  | *β_A_ = β_C_ = 0* | 2.681 | 2 | 0.262 | 4172.795 |
|  | ***β_A_ = β_E_ = 0*** | **0.896** | **2** | **0.639** | **4171.010** |
|  | *β_C_ = β_E_ = 0* | 1.042 | 2 | 0.594 | 4171.156 |
|  | *β_A_ = β_C_ = β_E_ = 0* | 5.021 | 3 | 0.170 | 4173.135 |
|  |  |  |  |  |  |
| ***14*** | *ace β_A_ β_C_ β_E_ β_M_* | -- | -- | -- | 2769.711 |
|  | *β_A_ = 0* | 3.975 | 1 | *0.046 | 2771.686 |
|  | *β_C_ = 0* | 3.653 | 1 | *0.056 | 2771.364 |
|  | *β_E_ = 0* | 1.718 | 1 | 0.190 | 2769.429 |
|  | *β_A_ = β_C_ = 0* | 5.748 | 2 | 0.056 | 2771.460 |
|  | *β_A_ = β_E_ = 0* | 4.012 | 2 | 0.135 | 2769.723 |
|  | *β_C_ = β_E_ = 0* | 4.500 | 2 | 0.105 | 2770.211 |
|  | ***β_A_ = β_C_ = β_E_ = 0*** | **5.751** | **3** | **0.124** | **2769.462** |

Model fit for twins with 7-year parental education and occupation. Bold rows show best fitting model as indicated by AIC.

* = significantly worse model fit as indicated by p-value
