# Supplementary material for: Socioeconomic Status (SES) and Children's Intelligence (IQ): In a UK-Representative Sample SES Moderates the Environmental, Not Genetic, Effect on IQ
Source: PLoS One. 2012 Feb 1;7(2):e30320. doi: 10.1371/journal.pone.0030320 (PMC3270016; doi:10.1371/journal.pone.0030320)
Supplement: Table S3 — Continous moderator model fit – SES index 3. Model fit for twins with 9-year family income at age 9. Bold rows show best fitting model as indicated by AIC. (DOC) [file pone.0030320.s003.doc]

**Table S3** Continuous moderator model fit for IQ by SES index 3 - family income at age 9

| ***Age*** | ***Model*** | ***Δ-2lnL*** | ***Δdf*** | ***p-value*** | ***AIC*** |
| --- | --- | --- | --- | --- | --- |
| ***9*** | *ace β_A_ β_C_ β_E_ β_M_* | -- | -- | -- | 2971.476 |
|  | *β_A_* = 0 | 0.088 | 1 | 0.767 | 2969.563 |
|  | *β_C_* = 0 | 3.950 | 1 | *0.047 | 2973.425 |
|  | *β_E_* = 0 | 0.124 | 1 | 0.724 | 2969.600 |
|  | *β_A_* = *β_C_* = 0 | 11.933 | 2 | *0.003 | 2979.409 |
|  | ***β_A_* = *β_E_* = 0** | **0.138** | **2** | **0.933** | **2967.614** |
|  | *β_C_* = *β_E_* = 0 | 5.061 | 2 | *0.080 | 2972.537 |
|  | *β_A_* = *β_C_* = *β_E_* = 0 | 12.181 | 3 | *0.007 | 2977.656 |
|  |  |  |  |  |  |
| ***10*** | *ace β_A_ β_C_ β_E_ β_M_* | -- | -- | -- | 2309.272 |
|  | *β_A_* = 0 | 0.902 | 1 | 0.342 | 2308.174 |
|  | *β_C_* = 0 | 0.808 | 1 | 0.369 | 2308.080 |
|  | *β_E_* = 0 | 0.579 | 1 | 0.447 | 2307.851 |
|  | *β_A_* = *β_C_* = 0 | 12.190 | 2 | *0.002 | 2317.462 |
|  | ***β_A_* = *β_E_* = 0** | **0.940** | **2** | **0.625** | **2306.212** |
|  | *β_C_* = *β_E_* = 0 | 2.517 | 2 | 0.284 | 2307.790 |
|  | *β_A_* = *β_C_* = *β_E_* = 0 | 12.680 | 3 | *0.005 | 2315.952 |
|  |  |  |  |  |  |
| ***12*** | *ace β_A_ β_C_ β_E_ β_M_* | -- | -- | -- | 2196.403 |
|  | *β_A_* = 0 | 0.262 | 1 | 0.609 | 2194.665 |
|  | *β_C_* = 0 | 0.802 | 1 | 0.371 | 2195.205 |
|  | *β_E_* = 0 | 0.005 | 1 | 0.943 | 2194.408 |
|  | *β_A_* = *β_C_* = 0 | 7.627 | 2 | *0.022 | 2200.029 |
|  | ***β_A_* = *β_E_* = 0** | **0.515** | **2** | **0.773** | **2192.918** |
|  | *β_C_* = *β_E_* = 0 | 0.988 | 2 | 0.610 | 2193.391 |
|  | *β_A_* = *β_C_* = *β_E_* = 0 | 9.540 | 3 | *0.023 | 2199.943 |
|  |  |  |  |  |  |
| ***14*** | ace *β_A_ β_C_ β_E_ β_M_* | -- | -- | -- | 1734.714 |
|  | *β_A_* = 0 | 1.565 | 1 | 0.211 | 1734.279 |
|  | *β_C_* = 0 | 3.091 | 1 | *0.079 | 1735.805 |
|  | *β_E_* = 0 | 2.634 | 1 | 0.105 | 1735.348 |
|  | *β_A_* = *β_C_* = 0 | 3.095 | 2 | 0.213 | 1733.808 |
|  | ***β_A_* = *β_E_* = 0** | **2.779** | **2** | **0.249** | **1733.493** |
|  | *β_C_* = *β_E_* = 0 | 4.618 | 2 | *0.099 | 1735.332 |
|  | *β_A_* = *β_C_* = *β_E_* = 0 | 5.814 | 3 | 0.121 | 1734.527 |

Model fit for twins with 9-year family income at age 9. Bold rows show best fitting model as indicated by AIC.

* = significantly worse model fit as indicated by p-value
